# 7-(6-Bromo­hex­yloxy)-4-methyl-2*H*-chromen-2-one

**DOI:** 10.1107/S1600536812020442

**Published:** 2012-05-12

**Authors:** Hui-Zhen Zhang, Qing-Xia Li, Ben-Tao Yin, Cheng-He Zhou

**Affiliations:** a Laboratory of Bioorganic & Medicinal Chemistry, School of Chemistry and Chemical Engineering, Southwest University, Chongqing 400715, People’s Republic of China

## Abstract

In the title mol­ecule, C_16_H_19_BrO_3_, all non-H atoms apart from the Br atom are approximately coplanar, with a maximum deviation of 0.242 (4) Å. The C—C—C—Br torsion angle is 66.5 (4)°.

## Related literature

For the pharmacological activity of coumarin compounds, see: Wu *et al.* (2009 ▶); Shi & Zhou (2011 ▶). For details of the synthesis, see: Shi *et al.* (2011 ▶). For a related structure, see: Zhang *et al.* (2011 ▶).
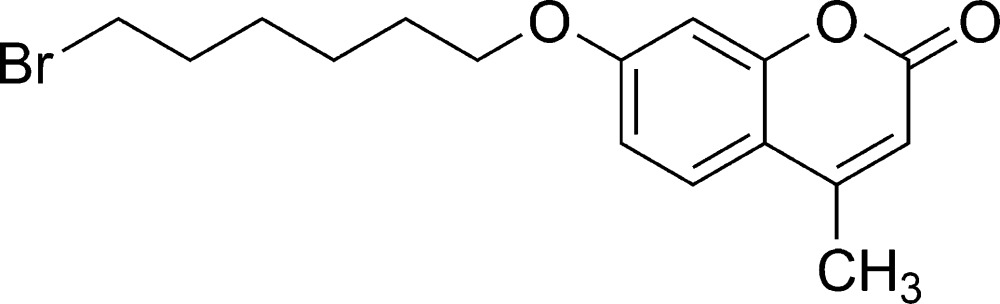


## Experimental

### 

#### Crystal data

C_16_H_19_BrO_3_
*M* *_r_* = 339.22
Monoclinic, 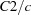

*a* = 15.681 (5) Å
*b* = 9.540 (3) Å
*c* = 22.104 (7) Å
β = 110.201 (6)°
*V* = 3103.3 (18) Å^3^
*Z* = 8
Mo *K*α radiation
μ = 2.65 mm^−1^
*T* = 296 K
0.22 × 0.18 × 0.15 mm

#### Data collection

Bruker APEXII CCD diffractometer
Absorption correction: multi-scan (*SADABS*; Bruker, 2009 ▶) *T*_min_ = 0.593, *T*_max_ = 0.692
8381 measured reflections
3051 independent reflections
1921 reflections with *I* > 2σ(*I*)
*R*_int_ = 0.028

#### Refinement

*R*[*F* ^2^ > 2σ(*F* ^2^)] = 0.042
*wR*(*F* ^2^) = 0.115
*S* = 1.01
3051 reflections
181 parameters
H-atom parameters constrained
Δρ_max_ = 0.48 e Å^−3^
Δρ_min_ = −0.53 e Å^−3^

### 

Data collection: *APEX2* (Sheldrick, 2008 ▶); cell refinement: *SAINT* (Sheldrick, 2008 ▶); data reduction: *SAINT*; program(s) used to solve structure: *SHELXS97* (Sheldrick, 2008 ▶); program(s) used to refine structure: *SHELXL97* (Sheldrick, 2008 ▶); molecular graphics: *SHELXTL* (Sheldrick, 2008 ▶); software used to prepare material for publication: *SHELXTL*.

## Supplementary Material

Crystal structure: contains datablock(s) global, I. DOI: 10.1107/S1600536812020442/lh5459sup1.cif

Structure factors: contains datablock(s) I. DOI: 10.1107/S1600536812020442/lh5459Isup2.hkl

Supplementary material file. DOI: 10.1107/S1600536812020442/lh5459Isup3.cml

Additional supplementary materials:  crystallographic information; 3D view; checkCIF report

## Figures and Tables

**Fig. 1.**
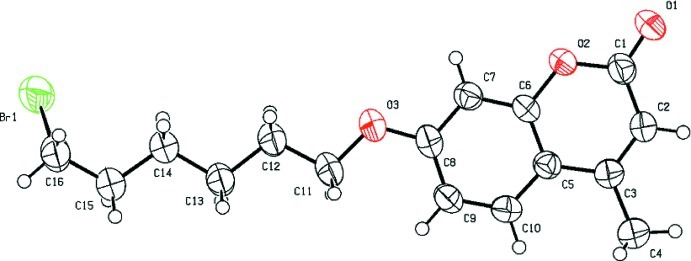
The molecular structure of (I), showing displacement ellipsoids drawn at the 50% probability level.

## supplementary crystallographic information

### Comment

Coumarin compounds are important in medicinal chemistry due to their extensive potential applications in antibacterial, antifungal, antiviral, anti-tubercular, anti-malarial, anticancer and anti-inflammatory fields (Wu, *et al.*, 2009; Shi & Zhou, 2011). Our interest is to develop novel coumarin compounds as antimicrobial agents and some structral related coumarin-triazoles have been reported as potential bioactive agents (Shi, *et al.*, 2011; Zhang, *et al.*, 2011). Herein, we report the crystal structure of the title compound (I).

The molecular structure of (I) is shown in Fig. 1. With the exception of the Br atom, all non-hydrogen atoms are approximately co-planar with a maximum deviation of 0.242 (4)Å (C16). The C14—C15—C16—Br1 torsion angle is 66.5 (4)Å.

### Experimental

Compound (I) was prepared according to the procedure of Shi & Zhou (2011). Single crystals were grown by slow evaporation of a solution of (I) in CHCl_3_ at room temperature.

### Refinement

H atoms were placed at calculated position with C—H = 0.93 Å (aromatic), 0.96 Å (methyl) and 0.97 Å (methylene). The *U*_iso_(H) values were set to 1.2U_eq_(C) or 1.5U_eq_(C_methyl_).

### Crystal data

**Table:**

C_16_H_19_BrO_3_	*F*(000) = 1392
*M**_r_* = 339.22	*D*_x_ = 1.452 Mg m^−^^3^
Monoclinic, *C*2/*c*	Mo *K*α radiation, λ = 0.71073 Å
Hall symbol: -C 2yc	Cell parameters from 2005 reflections
*a* = 15.681 (5) Å	θ = 2.6–22.1°
*b* = 9.540 (3) Å	µ = 2.65 mm^−^^1^
*c* = 22.104 (7) Å	*T* = 296 K
β = 110.201 (6)°	Block, colorless
*V* = 3103.3 (18) Å^3^	0.22 × 0.18 × 0.15 mm
*Z* = 8	

### Data collection

**Table:**

Bruker APEXII CCD diffractometer	3051 independent reflections
Radiation source: fine-focus sealed tube	1921 reflections with *I* > 2σ(*I*)
Graphite monochromator	*R*_int_ = 0.028
φ and ω scans	θ_max_ = 26.0°, θ_min_ = 2.5°
Absorption correction: multi-scan (*SADABS*; Bruker, 2009)	*h* = −19→19
*T*_min_ = 0.593, *T*_max_ = 0.692	*k* = −9→11
8381 measured reflections	*l* = −27→20

### Refinement

**Table:**

Refinement on *F*^2^	Primary atom site location: structure-invariant direct methods
Least-squares matrix: full	Secondary atom site location: difference Fourier map
*R*[*F*^2^ > 2σ(*F*^2^)] = 0.042	Hydrogen site location: inferred from neighbouring sites
*wR*(*F*^2^) = 0.115	H-atom parameters constrained
*S* = 1.01	*w* = 1/[σ^2^(*F*_o_^2^) + (0.0527*P*)^2^ + 2.7774*P*] where *P* = (*F*_o_^2^ + 2*F*_c_^2^)/3
3051 reflections	(Δ/σ)_max_ < 0.001
181 parameters	Δρ_max_ = 0.48 e Å^−^^3^
0 restraints	Δρ_min_ = −0.53 e Å^−^^3^

### Special details

**Table:**

Geometry. All e.s.d.'s (except the e.s.d. in the dihedral angle between two l.s. planes) are estimated using the full covariance matrix. The cell e.s.d.'s are taken into account individually in the estimation of e.s.d.'s in distances, angles and torsion angles; correlations between e.s.d.'s in cell parameters are only used when they are defined by crystal symmetry. An approximate (isotropic) treatment of cell e.s.d.'s is used for estimating e.s.d.'s involving l.s. planes.
Refinement. Refinement of *F*^2^ against ALL reflections. The weighted *R*-factor *wR* and goodness of fit *S* are based on *F*^2^, conventional *R*-factors *R* are based on *F*, with *F* set to zero for negative *F*^2^. The threshold expression of *F*^2^ > σ(*F*^2^) is used only for calculating *R*-factors(gt) *etc*. and is not relevant to the choice of reflections for refinement. *R*-factors based on *F*^2^ are statistically about twice as large as those based on *F*, and *R*- factors based on ALL data will be even larger.

### Fractional atomic coordinates and isotropic or equivalent isotropic displacement parameters (Å^2^)

**Table:**

	*x*	*y*	*z*	*U*_iso_*/*U*_eq_	
Br1	0.13272 (3)	0.92653 (5)	0.02243 (2)	0.0880 (2)	
C1	0.7117 (2)	−0.1670 (3)	0.24911 (16)	0.0537 (8)	
C2	0.6618 (2)	−0.2939 (3)	0.22745 (15)	0.0545 (8)	
H2A	0.6926	−0.3787	0.2379	0.065*	
C3	0.5727 (2)	−0.2965 (3)	0.19283 (15)	0.0513 (8)	
C4	0.5235 (3)	−0.4315 (3)	0.1708 (2)	0.0741 (11)	
H4A	0.5654	−0.5082	0.1847	0.111*	
H4B	0.4973	−0.4319	0.1246	0.111*	
H4C	0.4763	−0.4412	0.1889	0.111*	
C5	0.5242 (2)	−0.1647 (3)	0.17747 (14)	0.0442 (7)	
C6	0.57263 (19)	−0.0413 (3)	0.19839 (14)	0.0427 (7)	
C7	0.5329 (2)	0.0882 (3)	0.18675 (15)	0.0478 (7)	
H7A	0.5674	0.1687	0.2012	0.057*	
C8	0.4408 (2)	0.0971 (3)	0.15309 (15)	0.0514 (8)	
C9	0.3895 (2)	−0.0240 (3)	0.13091 (15)	0.0527 (8)	
H9A	0.3274	−0.0181	0.1083	0.063*	
C10	0.4319 (2)	−0.1512 (3)	0.14297 (15)	0.0528 (8)	
H10A	0.3978	−0.2316	0.1275	0.063*	
C11	0.3099 (2)	0.2471 (3)	0.11206 (17)	0.0602 (9)	
H11A	0.2765	0.1966	0.1348	0.072*	
H11B	0.2915	0.2118	0.0683	0.072*	
C12	0.2914 (2)	0.4009 (3)	0.11200 (19)	0.0612 (9)	
H12A	0.3306	0.4497	0.0932	0.073*	
H12B	0.3073	0.4324	0.1563	0.073*	
C13	0.1935 (2)	0.4414 (3)	0.07516 (16)	0.0557 (8)	
H13A	0.1779	0.4142	0.0303	0.067*	
H13B	0.1537	0.3911	0.0928	0.067*	
C14	0.1785 (2)	0.5971 (3)	0.07899 (17)	0.0584 (9)	
H14A	0.1936	0.6231	0.1239	0.070*	
H14B	0.2199	0.6466	0.0625	0.070*	
C15	0.0828 (2)	0.6447 (3)	0.04209 (17)	0.0579 (8)	
H15A	0.0713	0.6332	−0.0036	0.069*	
H15B	0.0407	0.5844	0.0533	0.069*	
C16	0.0638 (2)	0.7941 (4)	0.05454 (19)	0.0674 (10)	
H16A	0.0790	0.8079	0.1005	0.081*	
H16B	−0.0006	0.8128	0.0339	0.081*	
O1	0.79161 (16)	−0.1577 (2)	0.27985 (13)	0.0766 (8)	
O2	0.66442 (13)	−0.0436 (2)	0.23286 (10)	0.0503 (5)	
O3	0.40587 (14)	0.2292 (2)	0.14399 (12)	0.0649 (6)	

### Atomic displacement parameters (Å^2^)

**Table:**

	*U*^11^	*U*^22^	*U*^33^	*U*^12^	*U*^13^	*U*^23^
Br1	0.0703 (3)	0.0743 (3)	0.1032 (4)	0.0001 (2)	0.0092 (2)	0.0270 (2)
C1	0.0448 (19)	0.053 (2)	0.059 (2)	0.0053 (15)	0.0121 (16)	0.0118 (16)
C2	0.047 (2)	0.0450 (18)	0.063 (2)	0.0062 (15)	0.0084 (16)	0.0043 (15)
C3	0.050 (2)	0.0476 (18)	0.0523 (19)	0.0003 (14)	0.0122 (15)	−0.0034 (15)
C4	0.061 (2)	0.055 (2)	0.088 (3)	0.0001 (17)	0.003 (2)	−0.0139 (19)
C5	0.0413 (17)	0.0469 (17)	0.0416 (16)	0.0011 (13)	0.0107 (14)	−0.0022 (13)
C6	0.0331 (16)	0.0509 (18)	0.0416 (17)	0.0017 (13)	0.0097 (13)	0.0029 (13)
C7	0.0411 (17)	0.0485 (18)	0.0521 (19)	−0.0018 (14)	0.0141 (14)	0.0044 (14)
C8	0.0445 (18)	0.056 (2)	0.0533 (19)	0.0111 (15)	0.0160 (15)	0.0096 (15)
C9	0.0376 (17)	0.059 (2)	0.0545 (19)	0.0040 (15)	0.0065 (15)	−0.0024 (16)
C10	0.0442 (18)	0.0516 (19)	0.057 (2)	−0.0047 (14)	0.0096 (15)	−0.0085 (15)
C11	0.0435 (19)	0.064 (2)	0.070 (2)	0.0135 (16)	0.0152 (16)	0.0051 (18)
C12	0.048 (2)	0.057 (2)	0.077 (2)	0.0098 (15)	0.0192 (18)	0.0057 (17)
C13	0.0495 (19)	0.057 (2)	0.058 (2)	0.0099 (15)	0.0151 (16)	0.0016 (16)
C14	0.0501 (19)	0.055 (2)	0.065 (2)	0.0062 (15)	0.0135 (17)	0.0026 (16)
C15	0.052 (2)	0.056 (2)	0.063 (2)	0.0062 (16)	0.0154 (17)	0.0027 (16)
C16	0.057 (2)	0.065 (2)	0.080 (3)	0.0135 (17)	0.0220 (19)	0.0094 (19)
O1	0.0404 (14)	0.0630 (16)	0.104 (2)	0.0007 (11)	−0.0028 (14)	0.0119 (14)
O2	0.0358 (12)	0.0467 (12)	0.0610 (14)	−0.0013 (9)	0.0070 (10)	0.0049 (10)
O3	0.0437 (13)	0.0537 (13)	0.0888 (17)	0.0102 (10)	0.0120 (12)	0.0073 (12)

### Geometric parameters (Å, º)

**Table:**

Br1—C16	1.949 (4)	C9—H9A	0.9300
C1—O1	1.205 (4)	C10—H10A	0.9300
C1—O2	1.371 (4)	C11—O3	1.435 (4)
C1—C2	1.431 (4)	C11—C12	1.496 (4)
C2—C3	1.341 (4)	C11—H11A	0.9700
C2—H2A	0.9300	C11—H11B	0.9700
C3—C5	1.448 (4)	C12—C13	1.520 (4)
C3—C4	1.494 (4)	C12—H12A	0.9700
C4—H4A	0.9600	C12—H12B	0.9700
C4—H4B	0.9600	C13—C14	1.511 (4)
C4—H4C	0.9600	C13—H13A	0.9700
C5—C6	1.391 (4)	C13—H13B	0.9700
C5—C10	1.390 (4)	C14—C15	1.510 (4)
C6—O2	1.377 (3)	C14—H14A	0.9700
C6—C7	1.367 (4)	C14—H14B	0.9700
C7—C8	1.380 (4)	C15—C16	1.501 (5)
C7—H7A	0.9300	C15—H15A	0.9700
C8—O3	1.362 (4)	C15—H15B	0.9700
C8—C9	1.397 (4)	C16—H16A	0.9700
C9—C10	1.365 (4)	C16—H16B	0.9700
			
O1—C1—O2	116.6 (3)	O3—C11—H11B	110.4
O1—C1—C2	126.4 (3)	C12—C11—H11B	110.4
O2—C1—C2	117.0 (3)	H11A—C11—H11B	108.6
C3—C2—C1	123.2 (3)	C11—C12—C13	114.1 (3)
C3—C2—H2A	118.4	C11—C12—H12A	108.7
C1—C2—H2A	118.4	C13—C12—H12A	108.7
C2—C3—C5	118.5 (3)	C11—C12—H12B	108.7
C2—C3—C4	121.4 (3)	C13—C12—H12B	108.7
C5—C3—C4	120.1 (3)	H12A—C12—H12B	107.6
C3—C4—H4A	109.5	C14—C13—C12	111.6 (3)
C3—C4—H4B	109.5	C14—C13—H13A	109.3
H4A—C4—H4B	109.5	C12—C13—H13A	109.3
C3—C4—H4C	109.5	C14—C13—H13B	109.3
H4A—C4—H4C	109.5	C12—C13—H13B	109.3
H4B—C4—H4C	109.5	H13A—C13—H13B	108.0
C6—C5—C10	116.7 (3)	C15—C14—C13	114.2 (3)
C6—C5—C3	118.4 (3)	C15—C14—H14A	108.7
C10—C5—C3	124.9 (3)	C13—C14—H14A	108.7
O2—C6—C7	116.1 (3)	C15—C14—H14B	108.7
O2—C6—C5	121.1 (3)	C13—C14—H14B	108.7
C7—C6—C5	122.8 (3)	H14A—C14—H14B	107.6
C8—C7—C6	118.7 (3)	C16—C15—C14	114.2 (3)
C8—C7—H7A	120.6	C16—C15—H15A	108.7
C6—C7—H7A	120.6	C14—C15—H15A	108.7
O3—C8—C7	115.5 (3)	C16—C15—H15B	108.7
O3—C8—C9	124.1 (3)	C14—C15—H15B	108.7
C7—C8—C9	120.5 (3)	H15A—C15—H15B	107.6
C10—C9—C8	118.9 (3)	C15—C16—Br1	112.3 (2)
C10—C9—H9A	120.5	C15—C16—H16A	109.2
C8—C9—H9A	120.5	Br1—C16—H16A	109.2
C9—C10—C5	122.3 (3)	C15—C16—H16B	109.2
C9—C10—H10A	118.8	Br1—C16—H16B	109.2
C5—C10—H10A	118.8	H16A—C16—H16B	107.9
O3—C11—C12	106.6 (3)	C1—O2—C6	121.8 (2)
O3—C11—H11A	110.4	C8—O3—C11	118.9 (3)
C12—C11—H11A	110.4		
			
O1—C1—C2—C3	−179.4 (3)	C7—C8—C9—C10	−0.1 (5)
O2—C1—C2—C3	−0.1 (5)	C8—C9—C10—C5	1.1 (5)
C1—C2—C3—C5	−1.1 (5)	C6—C5—C10—C9	−1.3 (5)
C1—C2—C3—C4	179.5 (3)	C3—C5—C10—C9	178.8 (3)
C2—C3—C5—C6	1.1 (4)	O3—C11—C12—C13	175.4 (3)
C4—C3—C5—C6	−179.5 (3)	C11—C12—C13—C14	177.9 (3)
C2—C3—C5—C10	−179.0 (3)	C12—C13—C14—C15	178.8 (3)
C4—C3—C5—C10	0.5 (5)	C13—C14—C15—C16	170.5 (3)
C10—C5—C6—O2	−179.9 (3)	C14—C15—C16—Br1	66.5 (4)
C3—C5—C6—O2	0.0 (4)	O1—C1—O2—C6	−179.3 (3)
C10—C5—C6—C7	0.5 (4)	C2—C1—O2—C6	1.3 (4)
C3—C5—C6—C7	−179.5 (3)	C7—C6—O2—C1	178.3 (3)
O2—C6—C7—C8	−179.2 (3)	C5—C6—O2—C1	−1.3 (4)
C5—C6—C7—C8	0.3 (5)	C7—C8—O3—C11	−177.1 (3)
C6—C7—C8—O3	179.3 (3)	C9—C8—O3—C11	2.7 (5)
C6—C7—C8—C9	−0.6 (5)	C12—C11—O3—C8	177.3 (3)
O3—C8—C9—C10	−179.9 (3)		
